# The effect of n–π* electronic transitions on the N_2_ photofixation ability of carbon self-doped honeycomb-like g-C_3_N_4_ prepared *via* microwave treatment

**DOI:** 10.1039/d0ra00101e

**Published:** 2020-02-17

**Authors:** Xuelei Li, Jinfeng Bai, Jiaqi Li, Chao Li, Xiangyun Zhong, Shuping Deng

**Affiliations:** School of Chemical Engineering, University of Science and Technology Liaoning Anshan 114051 China baijinfeng2002@163.com; Department of Chemistry and Environmental Engineering, Yingkou Institute of Technology Bowen Road Yingkou 115014 China 15242339645@163.com

## Abstract

Light harvesting is an important part of the photocatalysis process. In this work, carbon self-doped honeycomb-like g-C_3_N_4_ with outstanding N_2_ photofixation ability was prepared *via* microwave treatment. XRD, N_2_ adsorption, UV-Vis, SEM, XPS, ESR and PL were used to characterize the as-prepared catalysts. Combining the carbon self-doping with microwave treatment, the n–π* transition was successfully stimulated. The remarkable red shift of absorption edge from 465 nm to near 600 nm was observed, leading to the obviously promoted visible light absorption. The synergy effect of carbon doping and microwave treatment also enhances the surface area and separation efficiency of electron–hole pairs. The as-prepared catalyst displays the highest NH_4_^+^ concentration of 5.3 mg L^−1^ g_cat_^−1^, over 11 times higher than that of neat g-C_3_N_4_, as well as excellent photocatalytic stability. DFT calculation was also used to further prove our point of view. This paper provides a new way for the construction of high efficiency photocatalysts.

## Abstract 2

Light harvesting is an important part of the photocatalysis process.

## Graphical abstract

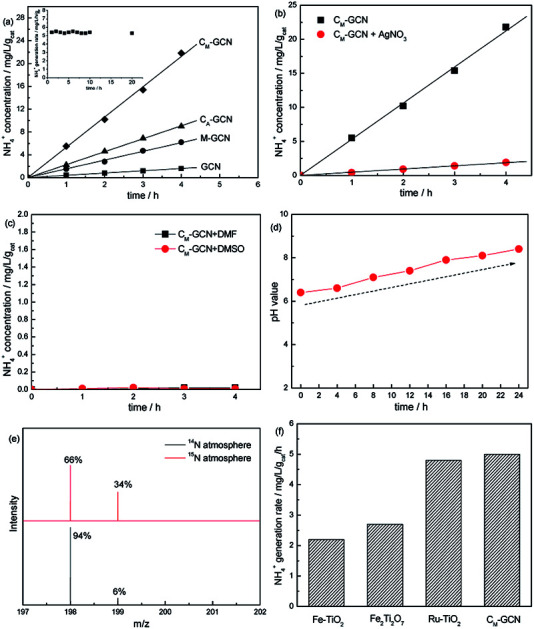

## Introduction

Artificial nitrogen fixation has become the second most important chemical reaction after photosynthesis. The traditional Haber–Bosch process is not in line with the standards of the contemporary chemical industry due to its high energy consumption, high risk and strong pollution characteristics. Therefore, finding a more energy-efficient, environmentally friendly and low-risk nitrogen fixation process is a hot topic in the scientific community.

Since the birth of life on Earth, it has survived mainly with the energy provided by the Sun. With the decline of fossil fuels, solar energy has become an important part of human energy use and has been continuously developed. Photocatalytic reaction is one of the ways to use solar energy.^1–4^ Its core mission is to find effective photocatalysts that can work stably for a long time under ultraviolet/visible light. So far, hundreds of photocatalysts have been reported one after another. Among them, graphite phase carbon nitride (g-C_3_N_4_) has attracted more and more researchers' attention due to its excellent performance, such as remarkable stability, fascinating electronic property and low-cost.^5,6^ However, g-C_3_N_4_ has a relatively large band gap, so that it can only absorb light with a wavelength of less than 460 nm. This makes the photocatalytic performance of g-C_3_N_4_ unsatisfactory.^7^ In order to improve the photocatalytic performance, researchers have used many methods to expand the photoresponse range of g-C_3_N_4_ based catalyst, including hetero-atomic doping and hetero-molecular doping.^8–11^ However, these exotic dopants also play as the recombination centres for photogenerated electron–hole, which is harmful to the photocatalytic performance.^12,13^

How to achieve the expansion of photoresponse range without addition the exotic dopants? The utilizing of lone pair electrons on N atoms is a effective measures.^14–16^ Theoretical calculations show that there are two feasible electronic transitions in g-C_3_N_4_, namely π–π* and n–π* transitions.^17^ The π–π* transition produces a strong absorption peak around 400 nm and an absorption boundary around 460 nm, as often shown in the UV-Vis spectrum. In contrast, the n–π* transition is an excitation of a lone pair on the N atom, and its corresponding absorption peak is around 500 nm. However, this n–π* transition is rarely reported although it can greatly expand the photoresponse range of the g-C_3_N_4_ based catalyst. This is due to that the lone pair of electrons of the N atom cannot be photoexcited in planar and symmetric heptazine units in g-C_3_N_4_.^18,19^ Therefore, an effective strategy is highly needed to achieve the excitation of such lone pair electrons in g-C_3_N_4_.

In order to achieve n–π* transition, heptazine units must be distorted to destroy the planar and symmetric g-C_3_N_4_. In this work, carbon self-doped honeycomb-like g-C_3_N_4_ with outstanding N_2_ photofixation ability was prepared *via* microwave treatment using supramolecular aggregates consisting of cyanuric acid, ethylene glycol and melamine as the polycondensation precursors. Combining the carbon self-doping with microwave treatment, the remarkable n–π* transition was stimulated. On the one hand, three precursors can self-assemble into supramolecular aggregates through multiple hydrogen bonds and π–π interactions.^20–22^ Such supramolecular aggregates can directly polymerize to obtain the modified g-C_3_N_4_ based catalyst. Ethylene glycol is not only used as a solvent to build supramolecular aggregates, but also simultaneously serves as a source for carbon doping, leading to the distortion of g-C_3_N_4_ construction.^23,24^ On the other hand, the microwave heating is very fast which can produce g-C_3_N_4_ in less than half hour. Lots of gases can be released during the microwave polymerization process of supramolecular aggregates. These rapid released gases create a special honeycomb-like three-dimensional structure of the g-C_3_N_4_ catalyst, leading to the further distortion of the structural unit in g-C_3_N_4_. In consequence, the n–π* transition becomes allowed in this work. DFT calculation was used to further prove our point of view.

## Experimental

### Preparation and characterization

5 g of melamine and 5 g of cyanuric acid were added into 150 mL of ethylene glycol (EG) at 60 °C under stirring. The white precipitate was obtained after cooling down. The obtained melamine–cyanuric acid–EG ternary complex was centrifuged, washed with ethanol, dried at 80 °C over night, and then transferred to an alumina crucible (25 mL). This crucible was put into a bigger alumina crucible and buried with the CuO powder. The sample was treated by microwave for 20 min in a normal microwave oven under Ar atmosphere, and denoted as C_M_-GCN. When ethanol was used to replace EG to synthesize C_M_-GCN, the obtained sample was denoted as M-GCN. In order to investigate the effect of microwave treatment on the photocatalytic performance, the melamine–cyanuric acid–EG ternary complex was annealed at 550 °C for 4 h under Ar atmosphere, the obtained sample was denoted as C_A_-GCN. For comparison, melamine was annealed at 550 °C for 4 h under Ar atmosphere, and denoted as GCN.

The structure characteristic of the sample was identified by X-ray diffraction (XRD, Rigaku D/max-2400). UV-Vis spectroscopy (JASCO-V-550) and scanning electron microscope (SEM, JSM 5600LV) were used to determine the optical property and morphology. X-ray photoelectron spectroscopy (XPS) measurement was carried out on Thermo Escalab 250 XPS system. Nitrogen adsorption–desorption isotherm was obtained using a Micromeritics 2010 analyzer. Electrochemical impedance spectra (EIS) was performed *via* an EIS spectrometer (EC-Lab SP-150, BioLogic Science Instruments). Electron Spin Resonance (ESR) and photoluminescence (PL) spectra were measured by digital X-band spectrometer (EMX-220, Bruker, USA) and FP-6300 equipment, respectively.

Material Studio 5.5 program (Accelrys, USA) was used in all the calculations. The generalized gradient approximation (GGA) with RPBE functional was utilized to describe the exchange and correlation potential and the basis set was set as double numerical plus polarization. A thermal meaning of 0.003 Ha was adopted to accelerate the convergence. The convergence criteria for geometry optimization and energy calculation were set as 1.0 × 10^−6^ Ha, 1.0 × 10^−5^ Ha, 0.002 Ha Å^−1^ and 0.005 Å for the tolerance of self-consistent field, energy, maximum force and maximum displacement, respectively.

### Photocatalytic reaction

The nitrogen photofixation reaction which performed in a double-walled quartz reactor was carried out according to previous work.^21,22^ 0.2 g of catalyst was added to 500 mL deionized water under stirring. Ethanol (0.789 g L^−1^) was added as a hole scavenger. A 250 W high-pressure sodium lamp (400 < *λ* < 800 nm) was used as the light source. 5 mL of the suspension were collected at given time intervals, and immediately centrifuged to separate the liquid samples. The concentration of NH_4_^+^ was measured using the Nessler's reagent spectrophotometry method (JB7478-87).^25,26^

## Results and discussion

**Fig. 1:**
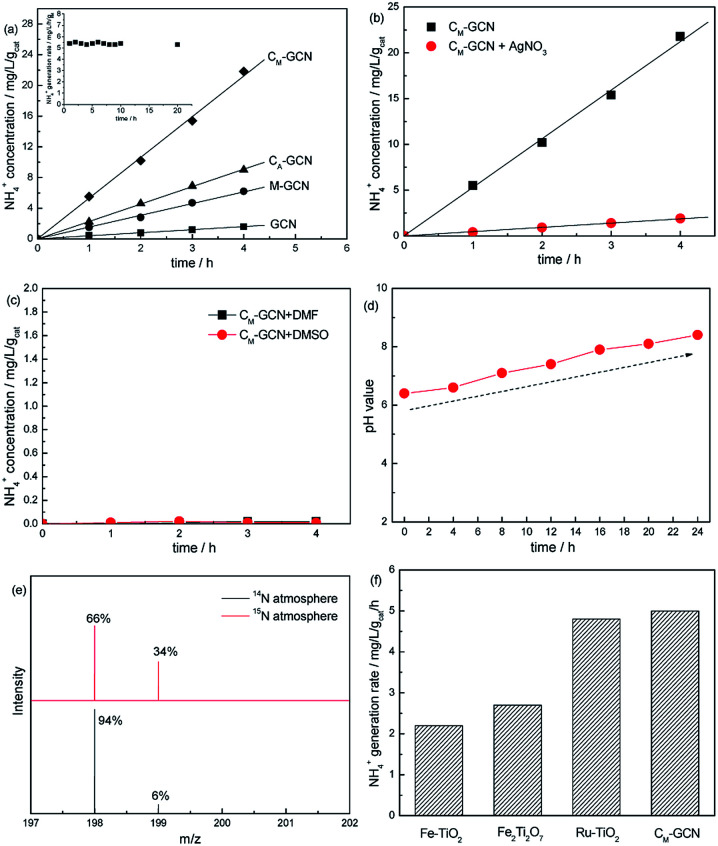
The N_2_ photofixation ability over as-prepared catalysts (a), N_2_ photofixation ability of C_M_-GCN using AgNO_3_ as the electron scavenger (b) or in aprotic solvents DMF and DMSO (c), N_2_ photofixation ability of C_M_-GCN under different pH value (d), the mass spectra of the indophenol prepared from different atmosphere (e) and the comparison of N_2_ photofixation ability of C_M_-GCN and other catalysts (f).

Fig. 1a shows the N_2_ photofixation ability over GCN, C_A_-GCN, M-GCN and C_M_-GCN. GCN shows very low NH_4_^+^ concentration, 0.48 mg L^−1^ g_cat_^−1^. For C_A_-GCN and M-GCN, the N_2_ photofixation abilities are obviously promoted to 2.3 and 1.5 mg L^−1^ g_cat_^−1^. C_M_-GCN displays the highest NH_4_^+^ concentration of 5.3 mg L^−1^ g_cat_^−1^, over 11 times higher than that of GCN, as well as the excellent photocatalytic stability. The N_2_ photofixation ability does not decrease after 20 h over C_M_-GCN (Fig. 1a inset). In Fig. 1b, when AgNO_3_ is added to trap the photogenerated electrons, the N_2_ photofixation ability of C_M_-GCN sharply decreases. This indicates that the photogenerated electrons are the main active species for the N_2_ photofixation. When DMF and DMSO are used as aprotic solvents to replace water, almost no NH_4_^+^ is formed (Fig. 1c). This hints water is necessary as the proton source in this reaction system. In Fig. 1d, before the N_2_ photofixation reaction, the pH value for the suspension is 6.4. This value gradually increases with the extension of the reaction time, and reaching 8.4 in 24 hours. This should be due to the consumption of H^+^ in this reaction system, as shown below:N_2_ + 6H^+^ + 6e^−^ ⇀ 2NH_3_NH_3_ + H_2_O ⇀ NH_3_·H_2_O ⇌ NH_4_^+^ + OH^−^

Isotopic labeling experiments are carried out to confirm the nitrogen source of NH_4_^+^. The N_2_ photofixation ability of C_M_-GCN was investigated under ^15^N isotope-labeled N_2_ (purity > 98%). The formed ^15^N labeled indophenol was analyzed by LC-MS and shown in Fig. 1e. Two strong indophenol anion signals obviously present at 198 and 199 *m*/*z* in LC-MS. This signal intensity is higher than the ^14^N : ^15^N natural abundance ratio, which confirms that N_2_ is the nitrogen source in this reaction system. Fig. 1f compares the N_2_ photofixation ability of C_M_-GCN and other catalysts reported in previous work.^26–28^ Obviously, C_M_-GCN exhibits much higher N_2_ photofixation ability than that of Fe–TiO_2_ and Fe_2_Ti_2_O_7_. Ru–TiO_2_ shows the comparable photocatalytic N_2_ fixation ability to that of C_M_-GCN. Considering the high price of Ru, C_M_-GCN has the best price/performance ratio.

**Fig. 2:**
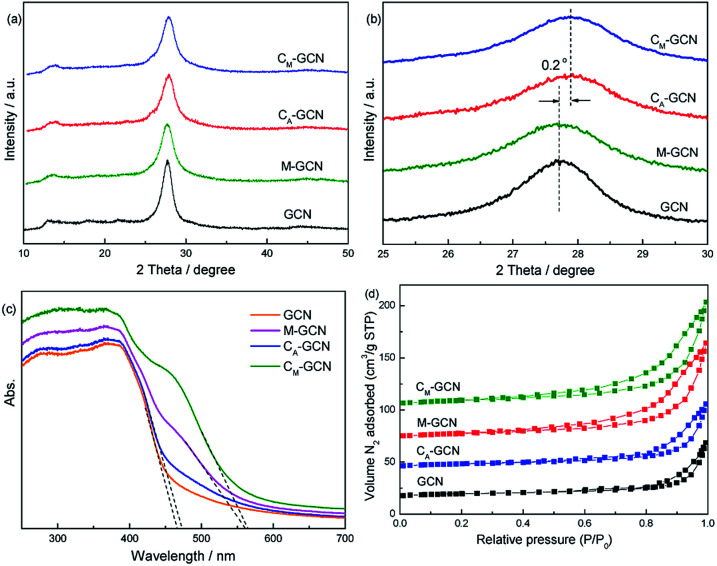
XRD patterns (a and b), UV-Vis spectra (c), and N_2_ adsorption–desorption isotherms (d) of as-prepared catalysts.

Fig. 2a and b shows the XRD patterns of GCN, C_A_-GCN, M-GCN and C_M_-GCN. A strong peak and a weak peak are observed for all the catalysts, which are assigned to the (002) and (100) crystal planes of g-C_3_N_4_.^29^ All the samples display the similar peak intensity, whereas C_A_-GCN and C_M_-GCN show a 0.2° shift toward higher 2*θ* value compared with GCN and M-GCN, as shown in magnified view (Fig. 2b). This should be due to the crystal lattice distortion after carbon doping, which is proved by previous work.^23,24^

UV-Vis spectra present the direct evidence for the n–π* electronic transition (Fig. 2c). GCN shows the absorption edge of ∼465 nm. For C_A_-GCN, the wavelength of absorption edge slight shifts to higher value, as well as the light absorption capacity from 450 to 600 nm a little increases. The band gap for the catalyst is calculated according to the following equation:^30^*E*_g_ = 1240/*λ*,where *λ* stands for the wavelength of the absorption edge (nm). The band gap energies for GCN and C_A_-GCN are calculated to be 2.67 and 2.65 eV, respectively. In the case of M-GCN and C_M_-GCN, a remarkable red shift of absorption edge from 465 nm to near 600 nm is observed. Besides that, the absorption peaks at ∼490 nm are obviously shown for M-GCN and C_M_-GCN. It is reported that this absorption peak is assigned to the n–π* electronic transition.^14^ This result indicates that the n–π* electronic transition is feasible in the g-C_3_N_4_ catalyst prepared by microwave treatment. C_M_-GCN shows much stronger absorption peaks at ∼490 nm than that of M-GCN. This suggests that carbon doping can distort the g-C_3_N_4_ lattice which is conducive to n–π* electronic transition. The band gaps for M-GCN and C_M_-GCN are 2.21 and 2.20 eV, respectively. In addition, it is interesting that the density for C_M_-GCN seems much smaller than that of other catalysts. With the same weight (0.2 g), the volume of C_M_-GCN is much larger than that of the other catalysts. This hints the structure of C_M_-GCN is fluffy, which may be caused by the special morphology. The N_2_ adsorption–desorption isotherms of GCN, C_A_-GCN, M-GCN and C_M_-GCN are shown in Fig. 2d. The isotherm of all the samples are of classical type IV, suggesting the presence of mesopores. The specific surface areas (*S*_BET_) of GCN is as low as 9.2 m^2^ g^−1^. For C_A_-GCN and M-GCN, their *S*_BET_ are promoted to 12.4 and 16.5 m^2^ g^−1^. In the case of C_M_-GCN, this value is greatly increased to 38.7 m^2^ g^−1^, which is probably due to its special morphology.

**Fig. 3:**
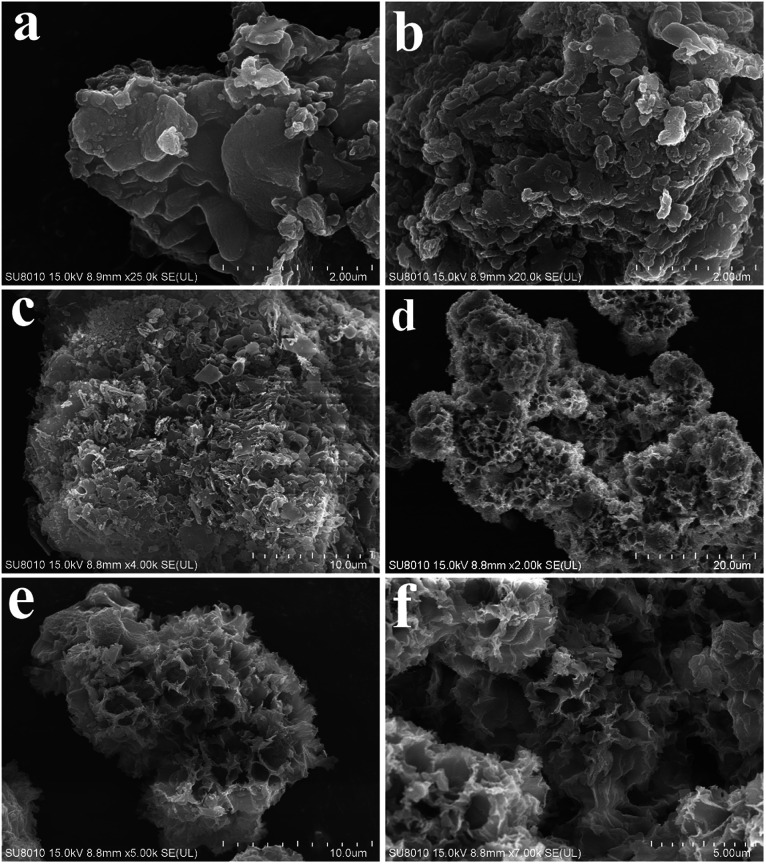
SEM images of GCN (a), C_A_-GCN (b), M-GCN (c) and C_M_-GCN (d–f).

The morphologies of GCN, C_A_-GCN, M-GCN and C_M_-GCN were tested by SEM and given in Fig. 3. It is shown that GCN has a graphite-like sheet structure (Fig. 3a). Those g-C_3_N_4_ nanosheets are stacked together to form a block structure. For C_A_-GCN, the sheet size significantly reduces compared with that of GCN (Fig. 3b). It can be seen in Fig. 3c that many irregular pores are formed in M-GCN. These g-C_3_N_4_ nanosheets are no longer superposed on each other flatly. In the case of C_M_-GCN (Fig. 3d–f), the three-dimensional honeycomb structure is clearly shown. Although the formation mechanism of such special morphology is still unclear, it can be concluded that both introducing dopant^16,31^ and microwave treatment^32,33^ have great effects on the catalyst morphology.

**Fig. 4:**
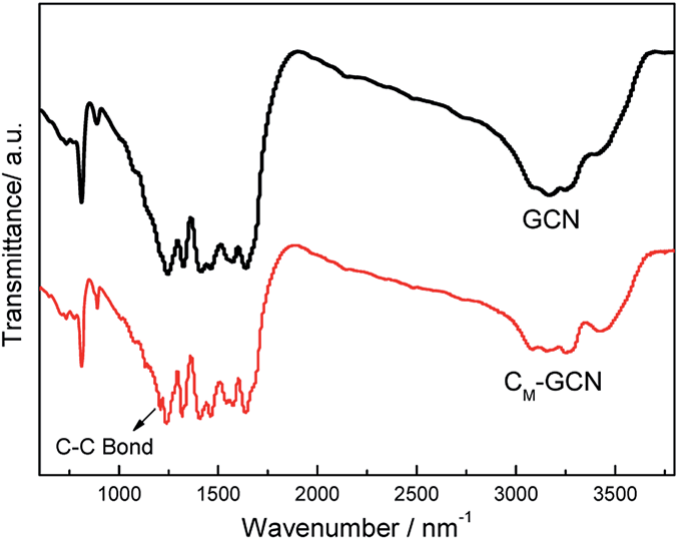
FT-IR spectra of GCN and C_M_-GCN.

The FT-IR spectra of GCN and C_M_-GCN are shown in Fig. 4. For GCN, a series of peaks in the range from 1200 to 1600 cm^−1^ are attributed to the typical stretching modes of CN heterocycles, while the sharp peak located at 810 cm^−1^ is assigned to the bending vibration of heptazine rings, which indicating the synthesized g-C_3_N_4_ is composed of heptazine units. The broad absorption band around 3200 cm^−1^ is originated from the stretching vibration of N–H bond, associated with uncondensed amino groups. In the case of C_M_-GCN, all the characteristic vibrational peaks of g-C_3_N_4_ are observed, suggesting that the structure of g-C_3_N_4_ is not changed. In addition, an obvious absorption peak appears at 1210 cm^−1^, which should be attributed to the C–C bond. This confirms that carbon doped into g-C_3_N_4_ lattice.

**Fig. 5:**
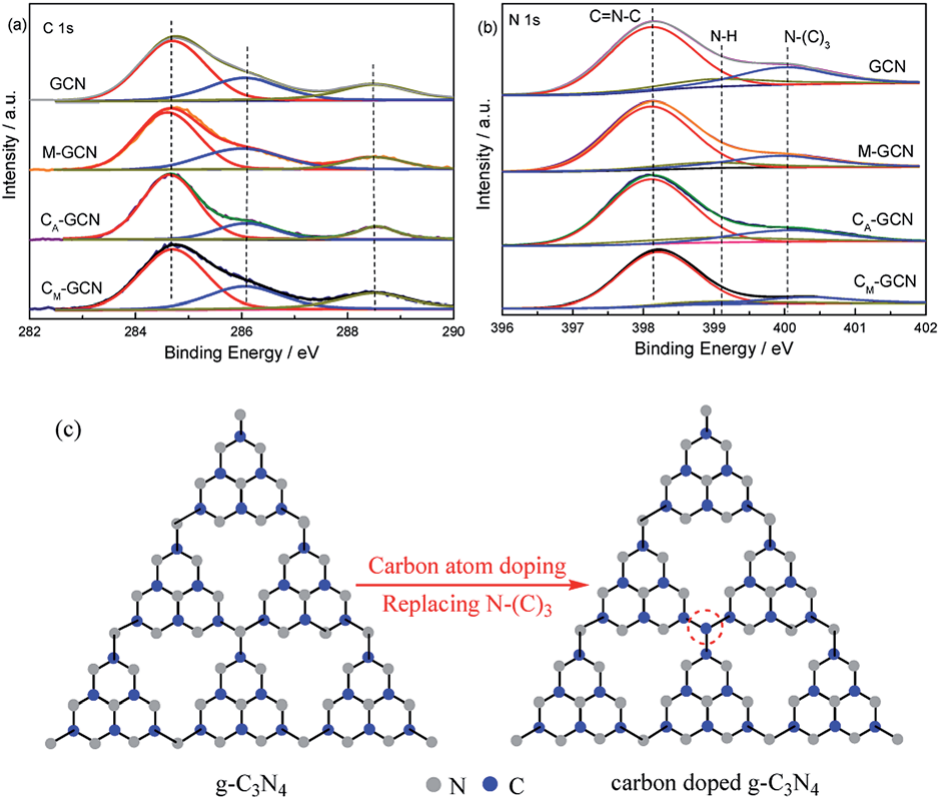
XPS spectra of GCN, C_A_-GCN, M-GCN and C_M_-GCN in the region of C 1s (a), N 1s (b) and the diagram of possible structure of g-C_3_N_4_ and carbon doped g-C_3_N_4_ (c).

The XPS spectra of GCN, C_A_-GCN, M-GCN and C_M_-GCN in the region of C 1s and N 1s regions are shown in Fig. 5. In C 1s region, the peak at 284.6 eV is the adventitious carbon. The peak around 286.2 and 288.6 eV are attributed to the terminal C–NH_*x*_ and sp^2^ hybridized C atom in the ring (N–C=N), respectively.^34^ No obvious difference among four catalysts is shown in C 1s region. However, the element analysis result shown in Table 1 indicates that the C/N ratio for GCN and M-GCN is 0.669. This value for C_A_-GCN and C_M_-GCN increases to 0.681 and 0.688, respectively. This result hints that carbon atoms have doped into the lattice of catalyst and the carbon source is ethylene glycol. In Fig. 5b, the N 1s spectra for all the samples can be divided into three peaks. The binding energies of 398.2 eV, 399.2 eV and 400.1 eV are assigned to the C–N=C (N_2C_), amino groups carrying hydrogen ((C)_2_–N–H) in connection with structural defects and incomplete condensation, and N–(C)_3_ (N_3C_) bond in g-C_3_N_4_, respectively.^34^ The binding energies for all the samples are the same, whereas the peak intensities vary greatly. The peak area ratio of N_2C_/N_3C_, calculated by the XPS data, is 3.68 for GCN. For M-GCN, microwave processing can distort the lattice of g-C_3_N_4_, leading to the higher N_2C_/N_3C_ ratio (4.15).^32,33^ The N_2C_/N_3C_ ratio for C_A_-GCN is 4.22, higher than GCN, which should be due to that the bridging N atoms are replaced by the doped carbon atoms,^35^ as shown in Fig. 5c. In the case of C_M_-GCN, the N_2C_/N_3C_ ratio further increases to 6.82. This clearly reveals that microwave treatment and carbon doping synergistically can cause the heptazine motifs to be further distorted. In addition, no binding energy in Cu 2p region is observed in XPS spectra. The ICP result also shows that no Cu is detected. Thus, the possibility of Cu doping is ruled out.

**Table 1:** Elemental composition of as-prepared catalysts

Sample	N (wt%)	C (wt%)	H (wt%)	C/N ratio
GCN	58.94	39.51	1.55	0.669
M-GCN	58.93	39.49	1.58	0.669
C_A_-GCN	58.39	39.77	1.84	0.681
C_M_-GCN	58.10	39.98	1.92	0.688

**Fig. 6:**
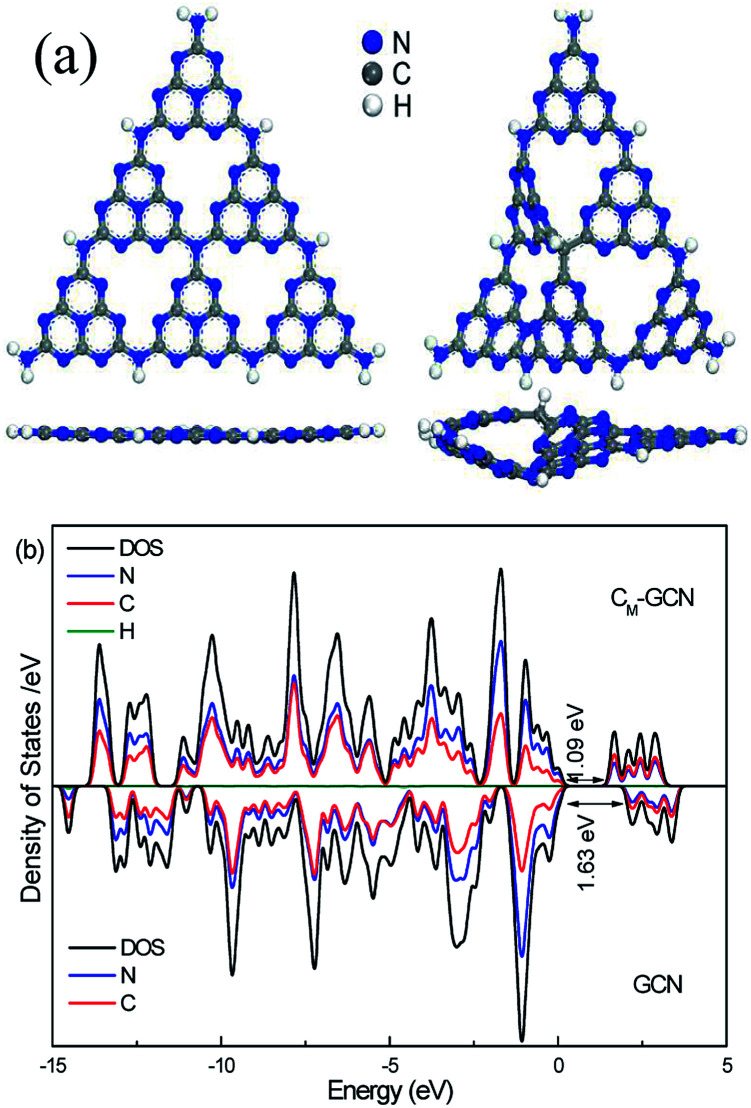
Optimized structure (a), density of states and partial density of states (b) of GCN and C_M_-GCN.

Fig. 6a shows the optimized structure of GCN and C_M_-GCN. Obviously, GCN displays the symmetrical planar structure. For C_M_-GCN, since the carbon atom is substituted for the nitrogen atom, a C–H bond is formed in order to achieve coordination saturation. The elemental composition shown in Table 1 indicates that the hydrogen content of C_M_-GCN obviously promote compared with GCN, which confirm the formation of C–H bond. A combination of carbon doping and additional C–H bond changes the g-C_3_N_4_ catalyst from a symmetrical planar structure to an asymmetrical non-planar structure, as shown in Fig. 6a. Thus, the n–π* transition becomes allowed for C_M_-GCN. This result theoretically supports the feasibility of the n–π* electronic transition shown in Fig. 2c. The electrical band structures of GCN and C_M_-GCN were also calculated using DFT. Fig. 6b displays the density of states and partial density of states of GCN and C_M_-GCN. It is obvious that the valence band maximum and the conduction band minimum of the catalysts are composed of C 2p and N 2p orbitals. C_M_-GCN shows much narrower band gap energy than that of GCN, which should be assigned to the n–π* electronic transition. This is consistent with the UV-Vis result.

**Fig. 7:**
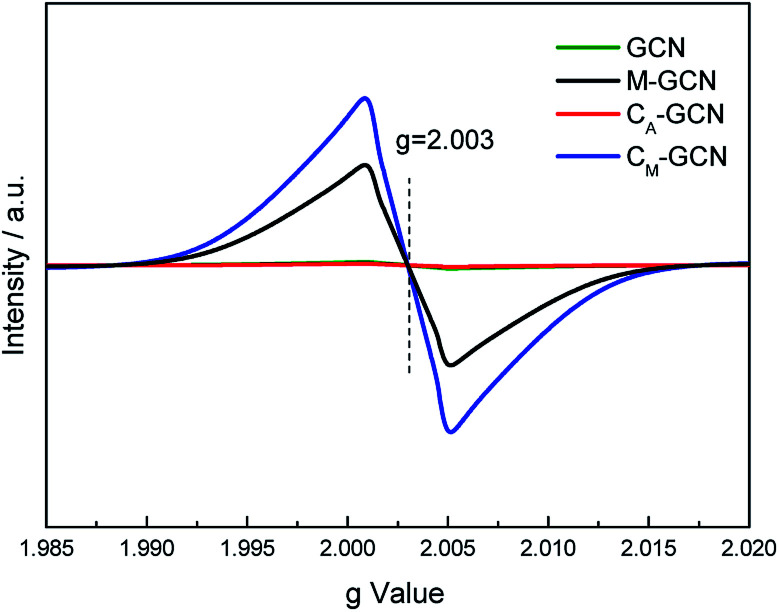
ESR spectra of GCN, C_A_-GCN, M-GCN and C_M_-GCN.

The EPR was carried out to further prove the n–π* electronic transition. As shown in Fig. 7, GCN and C_A_-GCN display no peak, hinting that almost no lone pair electrons exist in their g-C_3_N_4_ structure.^36,37^ M-GCN and C_M_-GCN show the distinct resonance signal at *g* = 2.003. This indicates that lots of localized lone pair electrons present in M-GCN and C_M_-GCN. These lone pair electrons can be excited through n–π* electronic transition.

**Fig. 8:**
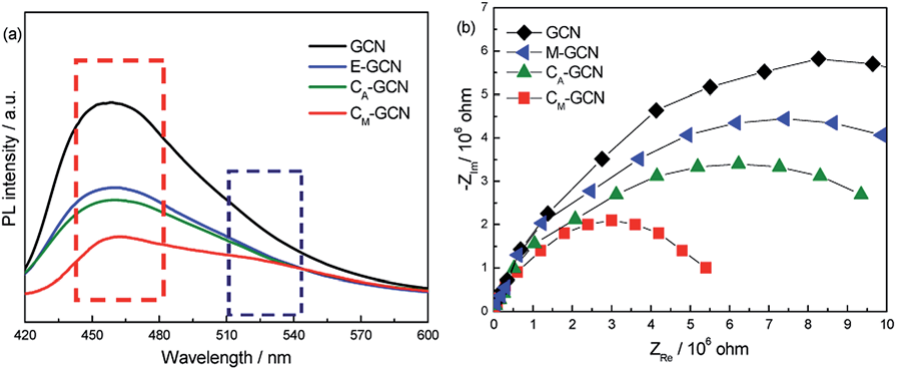
PL (a) and EIS (b) spectra of GCN, C_A_-GCN, M-GCN and C_M_-GCN.

The PL and EIS spectra of as-prepared catalysts are shown in Fig. 8. All the samples display the broad PL band around 460 nm (Fig. 7a), which can be attributed to the π–π* transition.^38,39^ GCN displays the most intense PL signal among all samples. M-GCN shows the decreased PL intensity compared with GCN, indicating that microwave treatment can promote the separation rate of electron–hole pairs. This is consistent with previous work.^32,33^ The PL intensity of C_A_-GCN is also lower than GCN. This should be due to that, when the bridging N atoms are substituted by the carbon atoms, the delocalized π bonds are formed between the hexatomic rings and the doping carbon atom. Such structure can enhance the electrical conductivity of g-C_3_N_4_ catalyst.^35^ In the case of C_M_-GCN, because of the synergy effect of doping and microwave treatment, its PL intensity is much lower than other samples, hinting its best separation efficiency. It is noted that, besides the PL band at 460 nm, a new peak around 520 nm is clearly shown for C_M_-GCN. This result confirms that the n–π* electronic transition occur in C_M_-GCN. Fig. 8b exhibits the EIS spectra of as-prepared catalysts. In general, the arc radius in the EIS spectra reflects the separation efficiency of electrons and holes.^40,41^ The smaller the arc radius, the higher the separation efficiency. The arc radius decreases in the order: GCN > M-GCN > C_A_-GCN > C_M_-GCN. This is consistent with the PL result that the most efficient electron–hole separation is C_M_-GCN.

**Fig. 9:**
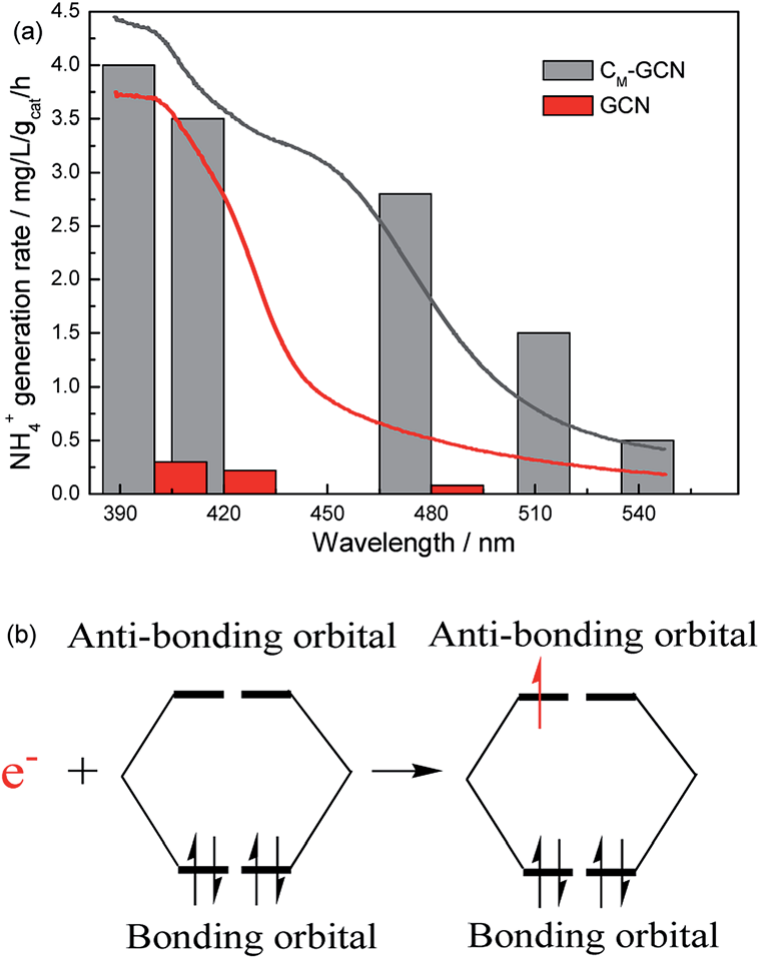
The N_2_ photofixation ability of GCN and C_M_-GCN under different wavelength (a) and the possible electron transfer process (b).

It is shown that the surface area and separation efficiency of electron–hole pairs are promoted by the synergy effect of doping and microwave treatment. In order to determine the effect of light absorption capacity of catalyst on N_2_ photofixation performance, the wavelength dependence of the NH_4_^+^ generation rate over GCN and C_M_-GCN was tested by using filters of different wavelengths (Fig. 9a). Interestingly, the N_2_ photofixation performance of GCN and C_M_-GCN is coincident with their optical absorption which is shown in UV-Vis spectra. C_M_-GCN displays the NH_4_^+^ generation rate of 0.5 mg L^−1^ g_cat_^−1^ h^−1^ even if the light with the wavelength below 550 nm is filtered out. However, the NH_4_^+^ generation rate of GCN becomes very low when the filter with 480 nm wavelength is used. This result clearly indicates that the main driving force for this N_2_ photofixation process is the ability to harvest the photons. After the n–π* transition, these photogenerated-electrons are transferred immediately from the catalyst to the adsorbed N_2_. Because the bonding orbitals of N_2_ molecule are occupied by four electrons, this photogenerated-electron has to occupy the anti-bonding orbitals, leading to the nitrogen activation (Fig. 9b). Thus, the nitrogen fixation performance is promoted.

**Fig. 10:**
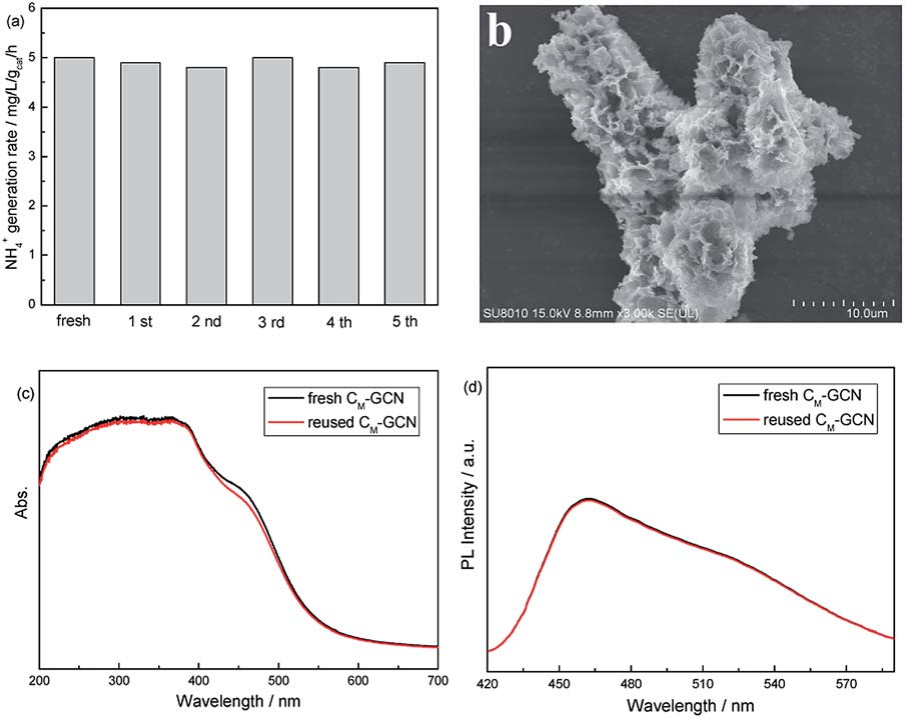
The photocatalytic stability of C_M_-GCN (a), the SEM image of reused C_M_-GCN (b), the UV-Vis spectra of fresh and reused C_M_-GCN (c), and the PL spectra of fresh and reused C_M_-GCN (d).

Fig. 10a shows the photocatalytic stability of C_M_-GCN. The NH_4_^+^ generation rate does not decrease after five cycles, confirming its good catalytic stability. The SEM image of reused C_M_-GCN is shown in Fig. 10b. The three-dimensional honeycomb structure is observed, hinting its excellent structural stability. Fig. 10c and d compares the UV-Vis and PL spectra of fresh and reused C_M_-GCN. No significant difference in UV-Vis and PL spectra between fresh and reused catalyst is observed. This indicates that the optical and electronic properties of C_M_-GCN does not change after five cycles.

## Conclusions

In this work, carbon self-doped honeycomb-like g-C_3_N_4_ with outstanding N_2_ photofixation ability was prepared *via* microwave treatment using supramolecular aggregates consisting of cyanuric acid, ethylene glycol and melamine as the polycondensation precursors. Ethylene glycol is not only used as a solvent to build supramolecular aggregates, but also simultaneously serves as a source for carbon doping. Microwave treatment can distort the structural unit of the catalyst. A combination of carbon doping and additional C–H bond further changes the g-C_3_N_4_ catalyst from a symmetrical planar structure to an asymmetrical non-planar structure, leading to the allowed n–π* transition for C_M_-GCN. The remarkable red shift of absorption edge from 465 nm to near 600 nm was observed, which cause the obviously promoted visible light absorption. The C_M_-GCN displays the highest NH_4_^+^ concentration of 5.3 mg L^−1^ g_cat_^−1^, over 11 times higher than that of neat g-C_3_N_4_, as well as the excellent catalytic and structural stability. The N_2_ photofixation ability of as-prepared catalysts under different wavelength indicates that the main driving force for this reaction is the ability to harvest the photons.

## Conflicts of interest

There are no conflicts to declare.

## Supplementary Material
